# Comparative analysis of whole genomes and transcriptomes of *Microsporum canis* from invasive dermatophytosis and tinea capitis

**DOI:** 10.1080/22221751.2023.2219346

**Published:** 2023-06-08

**Authors:** Ruojun Wang, Weixia Liu, Xiao Liu, Zhe Wan, G. Sybren de Hoog, Ruoyu Li, Yinggai Song

**Affiliations:** a Department of Dermatology and Venerology, Peking University First Hospital, Beijing, People’s Republic of China; b Research Center for Medical Mycology, Peking University, Beijing, People’s Republic of China; c National Clinical Research Center for Skin and Immune Diseases, Beijing, People’s Republic of China; d Beijing Key Laboratory of Molecular Diagnosis on Dermatoses, Beijing, People’s Republic of China; e Department of Dermatology, Heping Hospital Affiliated to Changzhi Medical College, Changzhi, People’s Republic of China; f Department of Dermatology and Venerology, Beijing Jishuitan Hospital, Beijing, People’s Republic of China; g Center of Expertise in Mycology of Radboud University Medical Center/Canisius Wilhelmina Hospital, Nijmegen, The Netherlands

**Keywords:** Dermatophytes, *Microsporum canis*, WGS, genomics, transcriptomics

## Abstract

Genomes of strains of the zoophilic dermatophyte *Microsporum canis* from invasive (disseminated and subcutaneous) and noninvasive (tinea capitis) infections were compared. Especially the disseminated strain showed significant syntenic rearrangements, including multiple translocations and inversions, and numerous SNPs and Indels in comparison to the noninvasive strain. In transcriptome analysis, both invasive strains were enriched for GO pathways related to components of the membrane, iron binding and heme binding, which possibly enables them to invade deeper into dermis and blood vessels. At 37 °C, invasive strains showed gene expression enriched for DNA replication, mismatch repair, N-glycan biosynthesis and ribosome biogenesis. The invasive strains were slightly less susceptible to multiple antifungal agents suggesting that acquired elevated drug resistance might be involved in the refractory disease courses. Patient with disseminated infection failed to respond to a combined antifungal treatment with itraconazole, terbinafine, fluconazole and posaconazole.

## Introduction

Dermatophytes are a monophyletic group of fungal pathogens that commonly affects the corneum stratum of the skin. Depending on the site of entry, infections variably present as tinea pedis, onychomycosis, tinea capitis or tinea corporis [1, 2]. *Microsporum canis* is a zoophilic dermatophyte that mainly affects dogs, cats, and other mammals, being carried asymptomatically in the fur. The fungus can be transmitted from animal to human hosts through direct contact, leading to clinical symptoms such as multifocal alopecia, scaling, or ringworm [3].

On very rare occasions, *M. canis* invades deeper into the dermis, causing pseudomycetoma-like infection of subcutaneous tissue, while even dissemination to internal organs [2] can be observed. Patients with chronic immunosuppression, specific genetic predispositions such as *CARD9*-related immunodeficiency, diabetes or lymphoproliferative disorders are at risk of developing deep infections [4]. Pseudomycetoma is a clinical variant of mycetoma comprising chronic dermal invasion; this condition is usually associated with long term tinea capitis and is refractory to antifungal therapy [5].

The host immune status is considered the main determinant in the development of invasive dermatophytoses. However, cases have been reported in immunocompetent individuals without any known predisposing factor, indicating that variance in the virulence of dermatophyte isolates also plays a role in invasive ability [2]. Several fungal virulence factors, such adhesins and proteases, account for pathogenesis of dermatophytes and contribute to establishment of infection [6]. However, the pathophysiological process of dermatophytes causing deeper infection of the dermis and internal organs is poorly understood. Different from other pathogenic fungi, dermatophytes favour growing at room temperature (30 °C) instead of human body temperature (37 °C), and 39 °C was found to be lethal to the organisms [7]. Invasion of dermatophytes is correlated with their growth and ribosomal biogenesis ability under stress, such as temperature shifts, osmotic stress, and exposure to antifungal drugs [6, 8].

In the present study, we sequenced the genomes and transcriptomes of two invasive *M. canis* strains collected from pseudomycetomata, and one noninvasive strain from tinea capitis. The whole genome sequences and gene expression profiles were compared between invasive and noninvasive strains and between different incubation temperatures to identify the potential genes and pathways responsible for deep infection by *Microsporum canis*.

## Results

### Clinical descriptions of *M. canis* strains

We added a three-letter indication behind each strain number (dis, sub, cap, i.e. disseminated, subcutaneous and tinea capitis, respectively) to enhance reading and distinction of the strains.

**Figure 1.**
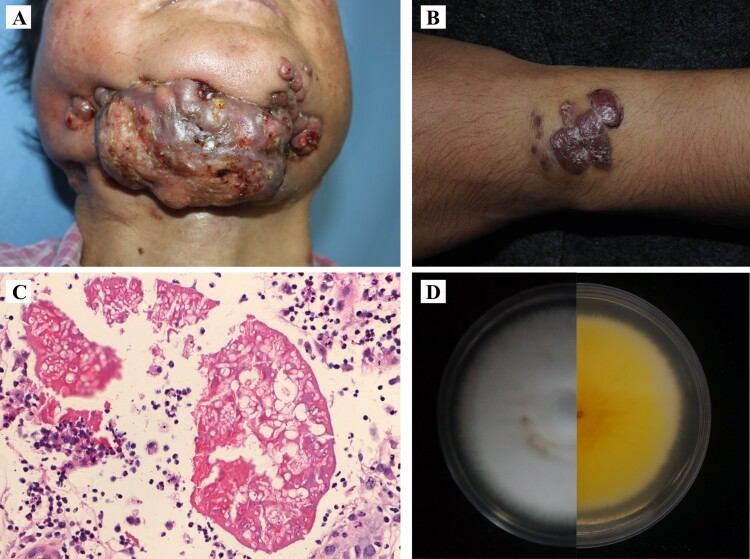
(A, B) Clinical presentations of case 1 and case 2. (C) Histopathological examination showing hyphal elements with Splendore-Hoeppli phenomenon. (D) Fungal culture showing a white and downy colony.

**Case 1, disseminated: BMU08102-dis** was collected from the skin of a 51-year-old female presenting with a 8-year history of numerous nodules and ulcerations involving the face, mandible, trunk, and extremities (Figure 1) [9]. She had been treated with itraconazole 400 mg daily for 2 years with improvement, but the lesions exacerbated after drug withdrawal. The patient was otherwise apparently healthy without use of immunosuppressive agents, diabetes, or other known underlying diseases. Laboratory examination revealed lymphocytopenia, including decreased CD4^+^ T cell count (13.74 / µl, normal range: 404–1612 / µl) and B cell count (32.22 / µl, normal range: 80–616 / µl). A test for HIV antibody was negative. We also evaluated the *CARD9* gene in this patient but found no mutations. Histopathological examination of the skin lesions revealed hyphal aggregates in the centre of moist, homogeneous eosinophilic material. Fungal culture of the pus and tissue grew white and downy colonies. Molecular identification was performed by sequencing the rDNA internal transcribed spacer (ITS), beta-tublin (*tub2*) and translation elongation factor 1-alpha (*tef1-α*) regions, all revealing 100% similarity to *M. canis*. However, macro- and microconidia remained absent or were distorted. Drug sensitivity tests showed that the strain was sensitive to terbinafine, azoles, and micafungin (Table 1). After surgical debridement of the mandibular lesion and pus, the patient was treated with terbinafine 250 mg daily and skin microwave therapy for 1 year without improvement of the lesions. During her hospitalization, she was started on a regimen of terbinafine 500 mg daily combined with intravenous fluconazole 400 mg per day for 12 days, followed by intravenous posaconazole 400 mg twice a day for 25 days. The abscesses and nodules reduced in the first 2 weeks, but no significant improvement was observed later. After discharge, she has treated with oral terbinafine 250 mg twice a day and itraconazole 200 mg twice a day. Patient failed to respond to a combined antifungal treatment and died of disseminated infection 10 years after onset of the infection.

**Table 1.** Drug sensitivity tests showing the minimum inhibitory concentrations (MIC, in µg/mL) of the strains.

	BMU08102-dis	BMU08359-sub	BMU11032-cap
Terbinafine	0.015	0.008	0.008
Fluconazole	4.0	4.0	8.0
Itraconazole	0.25	0.125	0.06
Posaconazole	0.06	0.06	0.06
Voriconazole	0.06	0.06	0.06
Amphotericin B	0.5	0.06	0.125
Micafungin	0.06	0.03	0.03

**Case 2, subcutaneous: BMU08359-sub** was isolated from a previously healthy 19-years-old female who presented with a 4-year history of asymptomatic red papules and nodules on her left wrist [10]. The patient received intralesional steroid injections and an excisional surgery, but subcutaneous nodules arose at the same site. Histopathological examination of a specimen revealed abscess formation containing hyphal elements with Splendore-Hoeppli phenomenon and multinucleated giant cells. The cultured isolate was identified by DNA sequencing, which showed 100% similarity to *M. canis* for the three loci (ITS, *tub2* and *tef1-α*). Microscopic exanimation of the culture revealed absence of micro- and macroconidia. An evaluation of patient’s *CARD9* gene showed no mutations. Drug sensitivity tests showed that the strain was sensitive to most antifungal agents (Table 1). The patient was administered itraconazole 200 mg daily with improvement but showed relapse six months later. She underwent an excisional surgery and continued on itraconazole 400 mg daily for 1 year with complete remission.

**Case 3, tinea capitis: BMU11032-cap** was isolated from a previously healthy 18-month female child with tinea capitis. The child had daily contact with a cat, and she presented with an alopecia with white scales on the scalp; *M. canis* was isolated from the lesions and its identity was confirmed by DNA sequencing (100% similarity to *M. canis* for the three loci used in identification). Production of micro- and macroconidia could be observed under the microscopy. Drug sensitivity test results are shown in Table 1. The child was successfully treated with oral terbinafine 90 mg daily for 2 months.

### Fungal growth on different culture temperatures

**Figure 2.**
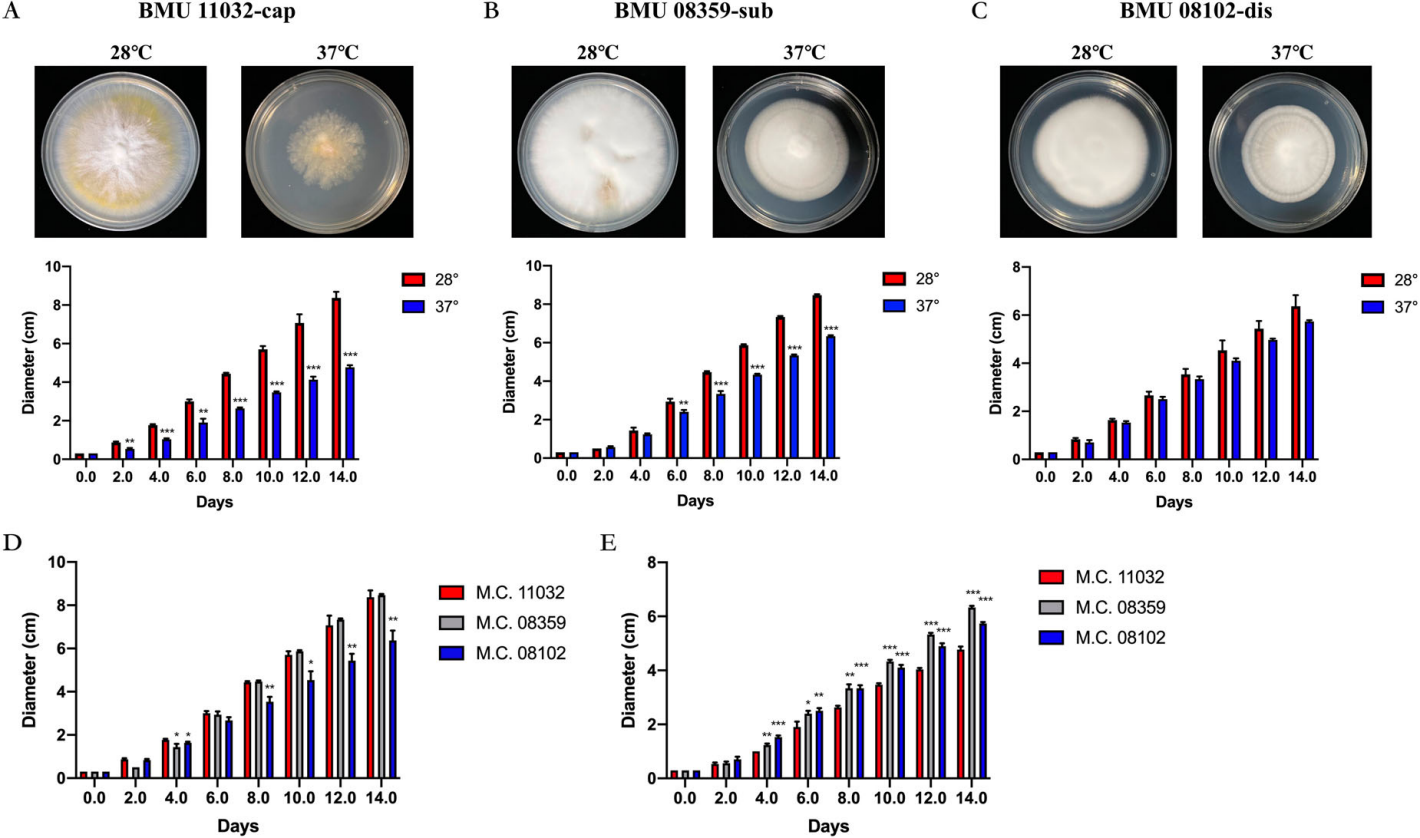
Colony diameters of BMU 11032-cap (A) BMU 08359-sub (B) and BMU08102-dis (C) in different culture temperatures at day 0, 2, 4, 6, 8, 10, 12 and 14. Comparison of growth diameters of the three *M. canis* strains at 28°C (D) and 37°C (E).

The diameters of BMU08102-dis, BMU 11032-cap and BMU 08359-sub were measured to illustrate temperature sensitivity of the three strains. BMU 11032-cap and BMU 08359-sub showed significantly decreased colony growth diameters at 37°C compared to those at 28°C since day 2 and day 6, respectively, whereas BMU08102-dis did not show differences in growth diameter (no significance) between various culture temperatures. When the three strains were compared, BMU08102-dis grew slower than BMU 11032-cap and BMU 08359-sub at 28°C. At 37°C, colonies of BMU 11032-cap showed significantly lower growth than BMU08102-dis and BMU 08359-sub (Figure 2).

### Sequence analysis and assembly

We sequenced the genomes of BMU11032-cap, BMU08359-sub and BMU08102-dis using a combination of long-read (PacBio) and short-read (Illumina) sequencing techniques. Second-generation sequencing was used as a supplementary and was combined with the third-generation sequencing using assembly software and parameter optimizations. The number of PacBio reads obtained for the three strains were 469,356 (7.13 Gb bases) for BMU08102-dis, 378,353 (5.51 Gb bases) for BMU08359-sub and 269,691 (3.26 Gb bases) for BMU11032-cap, suggesting larger genome sizes for the invasive strains.

The genome of the subcutaneous strain BMU08359-sub was 23,564,567 containing 7 contigs and the genome of the disseminated strain BMU08102-dis was 23,548,880 comprising 6 contigs. For the noninvasive strain BMU11032-cap, the genome was 23,577,342 bp organized into 7 contigs. Annotation of the strains yielded 6,455 (average length: 1,538) and 5,775 (average length: 1,398) protein-coding genes for the invasive strains BMU08102 and BMU08359, respectively, and 5058 genes (average length: 1,248) for the noninvasive strain BMU11032 (Table 2).

**Table 2.** Statistics of PacBio and Illumina sequencing.

Statistics of predicted gene	BMU08102 dis	BMU08359 sub	BMU11032 cap
Statistics of Illumina data			
Raw data (Mb)	1,148	1,177	1,172
Clean data (Mb)	1,000	1,000	1,000
Statistics of PacBio data			
Genome size (bp)	23,548,880	23,564,567	23,577,342
Number of bases (G)	7.13	5.51	3.26
GC%	47.27%	47.28%	47.27%
Gene number	6,455	5,775	5,058
Gene total length (bp)	9,928,566	8,074,218	6,310,779
Gene average length (bp)	1,538	1,398	1,248
GC% of predicted genes	51.16%	51.35%	47.27%
N50 length (bp)	18,860	17,975	15,615
N90 length (bp)	10,040	9,824	7,997
Number of contigs	6	7	7
N50 contig length (bp)	9,003,426	6,340,219	5,594,505
N90 contig length (bp)	1,650,681	2,902,373	2,908,379
Intergenic region length (bp)	13,620,314	15,490,349	17,266,563
Intergenic GC content	44.43%	45.17%	45.71%
tRNA	122	93	92
rRNA	29	38	25
sRNA	2	2	2

### Colinear analysis

**Figure 3.**
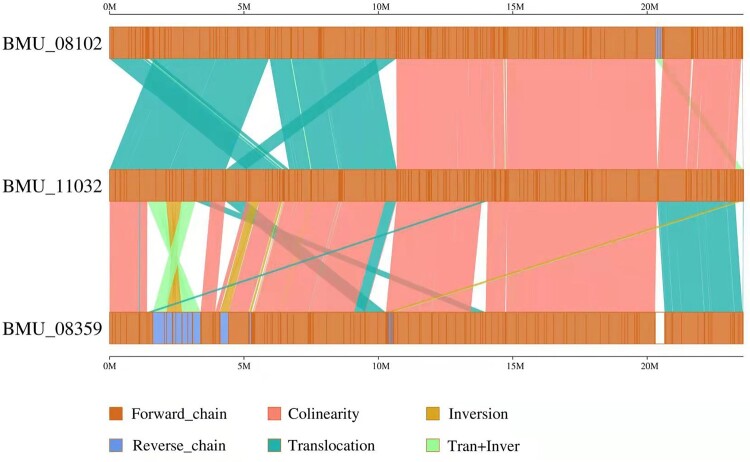
The linear genome comparisons of the three *M. canis* strains showing location of sequence rearrangements.

A total of 13,441 and 17,379 SNPs were identified for the invasive strains BMU08102-dis and BMU08359-sub compared to the noninvasive strain BMU11032-cap, respectively (Figure 3). The SNPs between BMU 08102-dis and BMU08359-sub were 2,908, indicating that the genomes of the invasive strains were more similar to each other than to the noninvasive strain. There were 269 (BMU08102-dis) and 1,061 (BMU08359-sub) synonymous SNPs, and 820 (BMU08102-dis) and 3,441 (BMU08359-sub) nonsynonymous SNPs compared to BMU11032-cap. The number of indels identified between the invasive strains was lower than that between the invasive strains and noninvasive strain. We found that substantial syntenic rearrangements, including inversions and translocations, between BMU08102-dis *vs*. BMU 11032-cap and BMU 08359-sub *vs.* BMU 11032-cap.

### Functional annotation

We performed the genome functional annotation and function prediction with seven databases (Table 3). The number of genes annotated in these databases was higher for the two invasive strains than for the noninvasive strain. Assignment with the GO database was performed to classify the functions of the predicted genes into three categories: biological process, cellular component, and molecular function. Annotation of the strains with the GO database showed higher than 60% of match efficiency for the two invasive strains (67.42% for BMU08102 and 65.13% for BMU08359), but only 37.33% of that for the noninvasive strain.

**Table 3.** Summary statistics of functional annotation of *M. canis* strains.

Number of predicted genes	BMU08102-dis	BMU08359-sub	BMU11032-cap
GO (n, %)	4,352 (67.42)	3,761 (65.13)	1,888 (37.33)
KEGG (n, %)	6,123 (94.86)	5,162 (89.39)	2,513 (49.68)
KOG (n, %)	1,800 (27.89)	1,435 (24.85)	676 (13.36)
NR (n, %)	6,289 (97.43)	5,394 (93.40)	2,633 (52.06)
Pfam (n, %)	4,352 (67.42)	3,761 (65.13)	1,888 (37.33)
SwissProt (n, %)	2,599 (40.26)	2,094 (36.26)	986 (19.49)
PHI (n, %)	1,040 (16.11)	830 (14.37)	390 (7.71)

The KEGG database, which was used to obtain an understanding of the biological pathways, was also aligned with more genes in the invasive strains than in the noninvasive strain. A total of 6,123 genes were included within 377 pathways for the strain BMU08102-dis, and 5,162 genes within 376 pathways for BMU08359-sub, and 2,513 genes within 356 pathways for the strain BMU11032-cap.

To investigate the pathogenicity of the three strains, their genomes were assigned to the PHI database. More lethal genes were annotated in the invasive strains than in the noninvasive strain, suggesting higher virulence of *M. canis* strains as being enhanced to cause invasive infections.

### Comparison of transcriptome profiles

**Figure 4.**
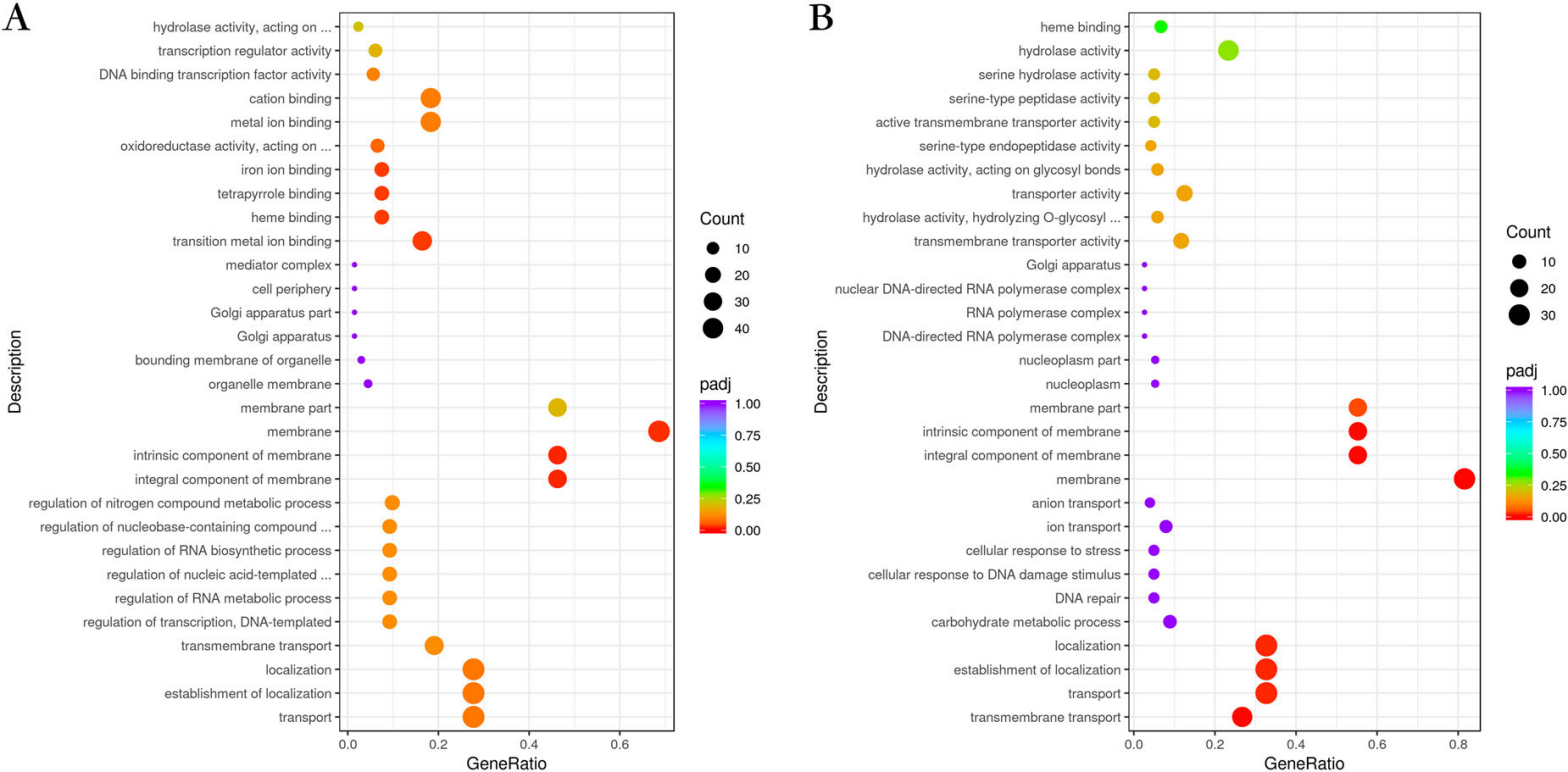
(A) Bubble plot showing the significantly enriched GO terms of upregulated genes for BMU08102-dis *vs*. BMU11032-cap. (B) The significantly enriched GO terms of upregulated genes for BMU08359-sub *vs*. BMU11032-cap.

Transcriptional profiles were analyzed and compared between the three *M. canis* strains (Figure 4). The upregulated genes for BMU08102-dis *vs*. BMU11032-cap were mainly in the cellular component and molecular function categories of the GO pathways, with components of membrane, iron ion binding, tetrapyrrole binding and heme binding being the most enriched subcategories. As for analysis of BMU08359-sub *vs.* BMU11032-cap, the upregulated genes of BMU08359-sub were significantly enriched in many GO subcategories of biological processes and cellular component, including transmembrane transport, establishment of localization and component of membrane.

### RNA-seq analysis between different culture temperatures

We analyzed the transcriptome profiles in *M. canis* strains cultured at 28°C and 37°C and found multiple differentially expressed genes (DEGs) under the compared conditions. There were 1,183 downregulated and 959 upregulated genes in BMU08102-dis 37°C *vs.* 28°C, 1,026 downregulated and 742 upregulated genes in BMU08359-sub 37°C *vs*. 28°C, and 290 downregulated and 287 upregulated genes in BMU11032-cap 37°C *vs*. 28°C.

**Figure 5.**
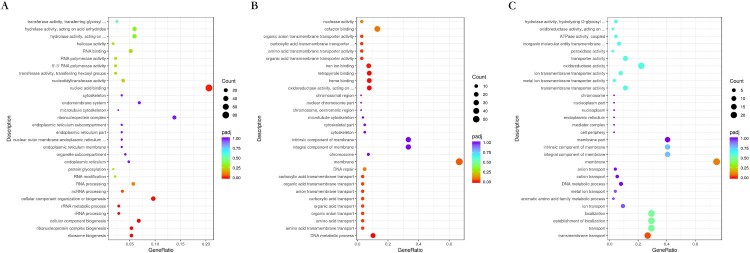
Bubble plot showing the significantly enriched KEGG terms of upregulated genes for BMU08102-dis (A), BMU08359-sub (B) and BMU11032-cap (C) 37°C *vs*. 28°C.

The GO enrichment analysis showed that the regulated genes in different degrees involved various biosynthetic processes for the three strains. For BMU08102-dis, the DEGs mainly act in ribosome biogenesis, ribonucleoprotein complex biogenesis and cellular component biogenesis. For BMU08359-sub, the DEGs are enriched in iron binding, cofactor binding, tetrapyrrole binding, and membrane, whereas transmembrane transport was the main enriched GO term in strain BMU11032-cap (Figure 5).

In KEGG enrichment analysis, comparison of invasive strains between 37°C *vs*. 28°C showed that the pathways of the upregulated genes of were mainly enriched in DNA replication, mismatch repair and N-glycan biosynthesis, while strain BMU11032-cap did not show these enrichments. The downregulated genes of all the three strains under 37°C mainly act in various metabolisms and the cellular, protein, amide, peptide and organic substance biosynthetic processes.

## Discussion

Skin infections by dermatophytes are common and usually present as superficial cutaneous mycoses targeting the stratum corneum. In immunosuppressed individuals or in patients with inherited immune deficiencies, dermatophytes may invade the dermis and even internal organs such as lymph nodes, bone, liver, and the brain [2]. In this study, we investigated the genome and transcriptome of two strains of *M. canis* which caused invasive infections in two otherwise healthy-appearing patients.

Invasive dermatophytosis can be divided into four categories according to their clinical and histological features: Majocchi’s granuloma, deeper dermal dermatophytosis, pseudomycetoma and disseminated infection [11]. Majocchi’s granuloma, which is characterized by suppurative folliculitis caused by disruption of hair follicles, is the most common type of deeper invasions of dermatophytes [12]. Deeper dermal dermatophytosis is characterized by extensive dermal and subcutaneous tissue invasion with a more severe disease course and mainly affects immunocompromised patients. It may present as granulomas, abscesses, lymphedema or draining sinuses [13]. Pseudomycetoma is a more severe variation of deeper dermal dermatophytosis, related to long-term superficial dermatophytosis and often affecting the scalp. It is characterized by the presence of microcolonies of the etiological organism with a surrounding eosinophilic matrix [14]. In very severe cases, dermatophytes can spread to lymph nodes and adjacent organs or cause life-threatening disseminated infection. This course of infection deviates considerably from mostly asymptomatic occurrence in feline fur, mostly leading to superficial tinea capitis upon transmission to human patients.

In our study, one of the patients with invasive dermatophytosis (BMU08359-sub) was immunocompetent, without any history of hyperglycemia, use of immunosuppressed agents, HIV infection or other known constitutional problems, while the other (BMU08102-dis) had impaired lymphocyte function and adaptive cytokine production of unknown origin. The *CARD9* gene, which is known to be a predisposing factor for chronic superficial and invasive fungal infections, was tested in both patients but did not show any mutations [15]. The skin biopsy specimens collected from both patients revealed Splendore-Hoeppli phenomenon suggesting a diagnosis of chronic deep infection. Although some cases of pseudomycetoma occurred in immunocompetent patients, disseminated disease has only been reported in three immunocompromised patients, all of whom underwent long-term administration of corticosteroids prior to the infection [12, 16]. Tissue invasion offers entirely different conditions for fungal growth compared to asymptomatic residence in mammal fur. Thus, we assume that the invasive *M. canis* strains might have some particularities in their genome or in gene expressions. The genomes of the two invasive *M. canis* strains, especially BMU08102-dis, showed significant syntenic rearrangements, including multiple translocations and inversions, and numerous SNPs and Indels in compared to BMU11032-cap.

In transcriptome analysis, the invasive strains of *M. canis* are enriched for GO pathways related to components of the membrane, iron binding and heme binding. Several membrane transport proteins, including the major facilitator superfamily, sugar transporter, inorganic phosphate transporter, polyamine transporter, calcium ion transporter and efflux transporters are found to be enriched in strains BMU08102-dis and BMU08359-sub. These membrane transporters are essential for acquisition of iron and nutrients, and their elevated expression may indicate facilitation of fungal growth.

Though strain BMU08102-dis showed higher MIC values to itraconazole and terbinafine than BMU08359-sub and BMU11032-cap, all of the three *M. canis* strains should be regarded as susceptible to these antifungal drugs judging from previously proposed breakpoints [17]. The breakpoints of amphotericin for dermatophytes have not been determined since this drug is rarely used in dermatophytoses. We found that the MIC value of strain BMU08102-dis against amphotericin B was close to the breakpoints for yeasts (above 0.5-1 μg/ml) and filamentous fungi (above 2 μg/ml) [18, 19]. The recalcitrance against multiple antifungal agents in both patients with deep infections suggests that the elevated expression of drug resistance-associated genes might be involved in the refractory disease courses. Dermatophytes respond to antifungal compounds by modulating the expression of genes related to drug efflux, lipid metabolism, protein signalling, transport and secretion, and oxidative stress [20–24]. In comparison to the noninvasive strain, the invasive strains showed increased expression of multiple efflux pump genes. However, whether the overexpression of efflux transporters detected in the invasive strains BMU08102-dis and BMU 08359-sub is caused by natural mutations or induced by long-term use of antifungal agents remains as yet unclear.

Iron, which plays a catalytic role as cofactor in many essential enzymes, is an indispensable element for living organisms [25]. However, iron acquisition in the host is challenging for pathogenic fungi because it is usually unavailable due to iron-binding proteins in the blood. The level of iron is even lower in the human stratum corneum, which is the preferred infection site of dermatophytes. Heme iron, mainly derived from hemoglobin, is a major source of iron for invasive microbial pathogens. Several fungi, such as *C. albicans*, have been found to be able to capture and extract heme from hemoglobin and utilize it as an iron source [26]. It may be speculated that the increased ability of iron and heme binding in the invasive *M. canis* isolates enables them to invade deeper into the dermis and vessels to survive in the living tissue.

The rarity of invasive dermatophytosis is mainly due to the unsuitable temperature of the human body for the growth of dermatophytes. The optimal temperature for dermatophytes is around 25°C, their development being suppressed at higher temperatures [27]. To analyze the potential mechanisms of the temperature tolerance of the invasive strains, we compared the gene expression profiles of the three *M. canis* strains between 28 and 37 °C. When cultured at 37°C, gene expression in both strains was enriched for DNA replication, mismatch repair, N-glycan biosynthesis and ribosome biogenesis compared to 28°C; this phenomenon was not observed in the noninvasive strain. These results indicate that the elevated proliferation ability and decreased metabolic processes of the invasive strains is probably associated with their deeper invasion into the dermis.

In this study, we demonstrated significant genomic rearrangement between invasive and noninvasive strains of *M. canis*. The transcriptome analysis showed that the invasive strains are characterized by several upregulated genes acting in iron and heme binding, and elevated DNA replication and mismatch repair. This study identifies gene families and pathways that can help our understanding of how *M. canis* cause invasive and chronic infections. Further studies focusing on the roles and effects of specific genes can help to investigate the mechanisms of invasive dermatophytosis.

## Materials and methods

### Sample collection, fungal culture and identification

Three clinical isolations of *M. canis* were analyzed. BMU08102-dis was isolated from a patient with disseminated dermatophytosis, BMU08359-sub was isolated from a patient with localized deep dermatophytosis, and BMU11032-cap was isolated from a patient with tinea capitis. For DNA extraction, the strains were cultured on Sabouraud’s Glucose Broth (SGB) for 7 days at 28°C prior to mycelial collection; and for RNA extraction, the strains were cultured at 28 and 37°C for 7 days.

The molecular identification was performed by sequencing the ITS (ITS1-F: 5’- TCCGTAGGTGAACCTGCGG-3’; ITS4-R: 5’-TCCTCCGCTTATTGATATGC-3’), beta-tubulin (Bt2a: 5’-GGTAACCAAATCGGTGCTGCTTTC-3’; Bt2b: 5’-ACCCTCAGTGTAGTGACCCTTGGC-3’) and TEF1-α (Derm-F: 5’-CACATTAACTTGGTCGTTATCG-3’; Derm-R: 5’-CATCCTTGGAGATACCAGC-3’) regions of rDNA.

### Fungal culture at different temperatures

*M. canis* strains were grown on a potato dextrose agar (PDA) at 28°C. We used biopsy punches that were 3 mm in diameter to obtain fungi, and then inoculated on the centre of PDA media. The strains were cultured at 28°C and 37°C and the diameter of each colony was measured every other day for 14 days. Experiments were performed in triplicate.

### Antifungal susceptibility testing

Antifungal susceptibility testing was conducted in accordance with the Clinical and Laboratory Standards Institute (CLSI) M38-A3 microbroth dilution method [28]. The tested drugs, including terbinafine, fluconazole, itraconazole, posaconazole, voriconazole, amphotericin B, and micafungin, were prepared following CLSI protocols.

MICs were read after incubation at 35°C after 7 days. Endpoint MICs were determined when approximately 80% inhibition was reached for azoles, terbinafine and micafungin, and 100% growth inhibition was reached for amphotericin B compared to the drug-free control [17]. Quality control strain of *T. mentagrophytes* (ATCC MYA 4439) was included in the experiments.

### DNA and RNA extraction

Genomic DNA was extracted using the MOBIO Power Microbial Maxi DNA Isolation kit (MoBio Laboratories, Carlsbad, CA, USA). The harvested DNA was detected by the agarose gel electrophoresis and quantified by Qubit® 2.0 Fluorometer (Thermo Fisher Scientific, Waltham, MA, USA).

Total RNA was extracted from cultures ground in liquid nitrogen using TRIZOL reagent (Thermo Scientific) according to the manufacturer’s instructions, followed by two phenol (pH 4.6)-chloroform-isoamyl alcohol (25:24:1) extraction steps and two chloroform extraction steps after the initial TRIZOL-chloroform phase separation [29]. RNA integrity was assessed using the RNA Nano 6000 Assay Kit of the Bioanalyzer 2100 system (Agilent Technologies, CA, USA).

### Library construction and PacBio sequencing

For Pacific Biosciences sequencing, libraries were constructed with an insert size of 20 kb using the SMRT bell TM Template kit, version 1.0. Briefly, the process was that fragment and concentrate DNA, repair DNA damage and ends, prepare blunt ligation reaction, purify SMRTbell Templates with 0.45X AMPure PB Beads, size-selection using the BluePippin System, repair DNA damage after size-selection. At last, the library quality was assessed on the Qubit® 2.0 Fluorometer (Thermo Scientific) and detected the insert fragment size by Agilent 2100 (Agilent Technologies). A total amount of 1μg DNA per sample was used as input material for the DNA sample preparations. Sequencing libraries were generated using NEBNext® UltraTM DNA Library Prep Kit for Illumina (NEB, USA) following manufacturer’s recommendations and index codes were added to attribute sequences to each sample. Briefly, the DNA sample was fragmented by sonication to a size of 350 bp, then DNA fragments were end-polished, A-tailed, and ligated with the full-length adaptor for Illumina sequencing with further PCR amplification. At last, PCR products were purified (AMPure XP system) and libraries were analysed for size distribution by Agilent2100 Bioanalyzer and quantified using real-time PCR. The whole genome of BMU08102, BMU11032 and BMU08359 was sequenced using PacBio Sequel platform at the Beijing Novogene Bioinformatics Technology Co., Ltd.

In addition to the PACBIO sequencing, we also performed Illumina paired-end sequencing for the assembly correction using Illumina NovaSeq PE150. A total amount of 1 μg DNA per sample was used as input material for the DNA sample preparations. Sequencing libraries were generated using NEBNext® UltraTM DNA Library Prep Kit for Illumina (NEB, USA) following manufacturer’s recommendations and index codes were added to attribute sequences to each sample. Briefly, the DNA sample was fragmented by sonication to a size of 350 bp, then DNA fragments were end-polished, A-tailed, and ligated with the full-length adaptor for Illumina sequencing with further PCR amplification. At last, PCR products were purified (AMPure XP system) and libraries were analyzed for size distribution by Agilent2100 Bioanalyzer and quantified using real-time PCR.

### Genome assembly

The bam files were converted from sequencing data into fastq format using samtools v1.3.1. Reads less than 5000 bp were filtered out. Mecat2 was used to perform correction, trimming and assembly, resulting in a draft assembled genome with a genome size setting of 35M [30]. Then, we performed 3 rounds of correction using minimap2-2.17 and racon-1.3.2, resulting in a consensus genome. The second-generation data were aligned with the consensus genome using bwa v0.7.12, sorted using samtools v1.3.1, and polished again using pilon-1.22.jar, resulting in a polished genome, which is the final result of the assembly process [31].

### Genome component prediction and gene function annotation

Genome component prediction included the prediction of the coding gene, repetitive sequences and non-coding RNA. The Augustus 2.7 program was used to retrieve the related coding gene. The interspersed repetitive sequences were predicted using the RepeatMasker v4.1.3-p1 (http://www.repeatmasker.org/). The tandem Repeats were analyzed by the TRF v4.10.0 (Tandem repeats finder). Transfer RNA (tRNA) genes were predicted by the tRNAscan-SE (v2.0). Ribosome RNA (rRNA) genes were analyzed by the rRNAmmer (v1.2). sRNA, snRNA and miRNA were predicted by BLAST (v2.2.28) against the Rfam database (v14.9 November 2022).

The software tools GeneMarkHMM (version 2), FGENESH (v2006), Augustus (v2.7), and SNAP (v2006-07-28), GlimmerHMM (v3.0.4) were used for selection of *ab initio* genes. Protein homology detection and intron resolution using the GeneWise software (v2.2.0) and the uniref90 non-redundant protein database. Alignment of known ESTs, full-length cDNAs, and most recently, Trinity RNA-Seq (v2.14.0) assemblies to the genome. PASA (v20130425beta) alignment assemblies based on overlapping transcript alignments from step. EVidenceModeler (EVM, v2.1.0) was used to compute weighted consensus gene structure annotations based on the above steps, and PASA (v20130425beta) was used to update the EVM consensus predictions, adding UTR annotations and models for alternatively spliced isoforms.

We used seven databases to predict gene functions: GO (Gene Ontology), KEGG (Kyoto Encyclopedia of Genes and Genomes), KOG (Clusters of Orthologous Groups), NR (Non-Redundant Protein Database databases), Pfam and Swiss-Prot. A whole genome Blast search (E-value less than 1e-5, minimal alignment length percentage larger than 40%) was performed against above seven databases. We used the PHI (Pathogen Host Interactions) database to analyze the pathogenicity and drug resistance analyses.

### Comparative genomics analysis

Comparative genomic analysis included the genomic synteny, the core genes and specific genes, SNP (Single Nucleotide Polymorphism), indel (insertion and deletion) and SV (Structural Variation) annotation, and genome visualization. (1) Genomic alignment between the two genomes were performed using the MUMmer (v3.23) and LASTZ (1.03.54) tools. Genomic synteny was analyzed based on the alignment results. (2) Core genes and specific genes were analyzed by the CD-HIT (v4.8.1) rapid clustering of similar proteins software with a threshold of 50% pairwise identity and 0.7 length difference cutoff in amino acid. (3) Blast was used to pairwise align all genes and eliminate the redundancy by solar and carried out gene family clustering treatment based on the alignment results with Hcluster_sg (v0.5.1) software. (4) SNP, indel and SV were found by the genomic alignment results among samples by the MUMmer and LASTZ we mentioned before.

### Transcriptome library preparation and sequencing

Total RNA was used as input material for the RNA sample preparations. The mRNA was purified from total RNA using poly-T oligo-attached magnetic beads. Fragmentation was carried out using divalent cations under elevated temperature in First Strand Synthesis Reaction Buffer (5X). First strand cDNA was synthesized using random hexamer primer and M-MuLV Reverse Transcriptase, then use RNaseH to degrade the RNA. Second strand cDNA synthesis was subsequently performed using DNA Polymerase I and dNTP. Remaining overhangs were converted into blunt ends via exonuclease/polymerase activities. After adenylation of 3’ ends of DNA fragments, Adaptor with hairpin loop structure were ligated to prepare for hybridization. In order to select cDNA fragments of preferentially 370∼420 bp in length, the library fragments were purified with AMPure XP system (Beckman Coulter, Beverly, USA). Then PCR was performed with Phusion High-Fidelity DNA polymerase, Universal PCR primers and Index (X) Primer. At last, PCR products were purified (AMPure XP system) and library quality was assessed on the Agilent Bioanalyzer 2100 system.

The clustering of the index-coded samples was performed on a cBot Cluster Generation System using TruSeq PE Cluster Kit v3-cBot-HS (Illumia) according to the manufacturer’s instructions. After cluster generation, the library preparations were sequenced on an Illumina Novaseq platform and 150 bp paired-end reads were generated.

### RNA-seq analysis

Reference genome and gene model annotation files were downloaded from genome website directly. Index of the reference genome was built using Hisat2 v2.0.5 and paired-end clean reads were aligned to the reference genome using Hisat2. FeatureCounts v1.5.0-p3 was used to count the reads numbers mapped to each gene. And then FPKM of each gene was calculated based on the length of the gene and reads count mapped to this gene. FPKM, expected number of Fragments Per Kilobase of transcript sequence per Millions base pairs sequenced, considers the effect of sequencing depth and gene length for the reads count at the same time, and is currently the most commonly used method for estimating gene expression levels.

Differential expression analysis of two conditions/groups (two biological replicates per condition) was performed using the DESeq2 R package (v1.20.0). The resulting *P*-values were adjusted using the Benjamini and Hochberg’s approach for controlling the false discovery rate. GO and KEGG enrichment analyses were performed by the clusterProfiler R package (v3.14.0), in which gene length bias was corrected. Genes with an adjusted *P*-value <0.05 found by DESeq2 were assigned as differentially expressed.

## Data Availability

All genomes and RNA-seq have been deposited in NCBI (accession number: PRJNA877796 and PRJNA877110).

## References

[1] Wu LC, Sun PL, Chang YT. Extensive deep dermatophytosis cause by Trichophyton rubrum in a patient with liver cirrhosis and chronic renal failure. Mycopathologia. 2013;176:457–462. 23982285 10.1007/s11046-013-9696-2

[2] Wang R, Huang C, Zhang Y, et al. Invasive dermatophyte infection: a systematic review. Mycoses. 2021;64:340–348. 33217082 10.1111/myc.13212

[3] Aneke CI, Otranto D, Cafarchia C. Therapy and antifungal susceptibility profile of Microsporum canis. J Fungi. 2018;4:107. 10.3390/jof4030107 PMC6162526 30189676

[4] Nazarian RM, Lilly E, Gavino C, et al. Novel CARD9 mutation in a patient with chronic invasive dermatophyte infection (tinea profunda). J Cutan Pathol. 2020;47:166–170. 31469433 10.1111/cup.13574

[5] Botterel F, Romand S, Cornet M, et al. Dermatophyte pseudomycetoma of the scalp: case report and review. Br J Dermatol. 2001;145:151–153. 11453926 10.1046/j.1365-2133.2001.04301.x

[6] Martinez-Rossi NM, Peres NT, Rossi A. Pathogenesis of dermatophytosis: sensing the host tissue. Mycopathologia. 2017;182:215–227. 27590362 10.1007/s11046-016-0057-9

[7] Lorincz AL, Sun SH. Dermatophyte viability at modestly raised temperatures. Arch Dermatol. 1963;88:393–402. 14051350 10.1001/archderm.1963.01590220025003

[8] Shore D, Zencir S, Albert B. Transcriptional control of ribosome biogenesis in yeast: links to growth and stress signals. Biochem Soc Trans. 2021;49:1589–1599. 34240738 10.1042/BST20201136 PMC8421047

[9] Song Y, Wang X, Li Q, et al. Fatal dermatophytic pseudomycetoma in a patient with non-HIV CD4 lymphocytopenia. Emerg Microbes Infect. 2023;12:2208685. 37128909 10.1080/22221751.2023.2208685 PMC10193896

[10] Wang R, Wang X, Li R. Image gallery: dermatophytic pseudomycetoma caused by Microsporum canis. Br J Dermatol. 2018;178:e228. 29595227 10.1111/bjd.16243

[11] Nir-Paz R, Elinav H, Pierard GE, et al. Deep infection by Trichophyton rubrum in an immunocompromised patient. J Clin Microbiol. 2003;41:5298–5301. 14605189 10.1128/JCM.41.11.5298-5301.2003 PMC262492

[12] Petrov I, Kempf W, Stoilova D, et al. Disseminated dermatophytic pseudomycetomas arising in an immunocompromised patient. Br J Dermatol. 2006;155:628–630. 16911295 10.1111/j.1365-2133.2006.07371.x

[13] Marconi VC, Kradin R, Marty FM, et al. Disseminated dermatophytosis in a patient with hereditary hemochromatosis and hepatic cirrhosis: case report and review of the literature. Med Mycol. 2010;48:518–527. 20092423 10.3109/13693780903213512

[14] Berg JC, Hamacher KL, Roberts GD. Pseudomycetoma caused by Microsporum canis in an immunosuppressed patient: a case report and review of the literature. J Cutan Pathol. 2007;34:431–434. 17448202 10.1111/j.1600-0560.2006.00628.x

[15] Queiroz-Telles F, Mercier T, Maertens J, et al. Successful allogenic stem cell transplantation in patients with inherited CARD9 deficiency. J Clin Immunol. 2019;39:462–469. 31222666 10.1007/s10875-019-00662-z

[16] Tirado-Gonzalez M, Ball E, Ruiz A, et al. Disseminated dermatophytic pseudomycetoma caused by Microsporum species. Int J Dermatol. 2012;51:1478–1482. 23171014 10.1111/j.1365-4632.2012.05550.x

[17] Jiang Y, Luo W, Verweij PE, et al. Regional differences in antifungal susceptibility of the prevalent dermatophyte Trichophyton rubrum. Mycopathologia. 2021;186:53–70. 33313977 10.1007/s11046-020-00515-z PMC7946697

[18] Maurya VK, Kachhwaha D, Bora A, et al. Determination of antifungal minimum inhibitory concentration and its clinical correlation among treatment failure cases of dermatophytosis. J Fam Med Prim Care. 2019;8:2577–2581. 10.4103/jfmpc.jfmpc_483_19 PMC6753804 31548935

[19] Wildfeuer A, Seidl HP, Paule I, et al. In vitro evaluation of voriconazole against clinical isolates of yeasts, moulds and dermatophytes in comparison with itraconazole, ketoconazole, amphotericin B and griseofulvin. Mycoses. 1998;41:309–319. 9861837 10.1111/j.1439-0507.1998.tb00344.x

[20] Zhang W, Yu L, Yang J, et al. Transcriptional profiles of response to terbinafine in Trichophyton rubrum. Appl Microbiol Biotechnol. 2009;82:1123–1130. 19234875 10.1007/s00253-009-1908-9

[21] Peres NT, Sanches PR, Falcao JP, et al. Transcriptional profiling reveals the expression of novel genes in response to various stimuli in the human dermatophyte Trichophyton rubrum. BMC Microbiol. 2010;10:39. 20144196 10.1186/1471-2180-10-39 PMC2831883

[22] Persinoti GF, de Aguiar Peres NT, Jacob TR, et al. RNA-sequencing analysis of Trichophyton rubrum transcriptome in response to sublethal doses of acriflavine. BMC Genom. 2014;15(Suppl. 7):S1. 10.1186/1471-2164-15-S7-S1 PMC4243288 25573029

[23] Mendes NS, Bitencourt TA, Sanches PR, et al. Transcriptome-wide survey of gene expression changes and alternative splicing in Trichophyton rubrum in response to undecanoic acid. Sci Rep. 2018;8:2520. 29410524 10.1038/s41598-018-20738-x PMC5802734

[24] Martinez-Rossi NM, Bitencourt TA, Peres NTA, et al. Dermatophyte resistance to antifungal drugs: mechanisms and prospectus. Front Microbiol. 2018;9:1108. 29896175 10.3389/fmicb.2018.01108 PMC5986900

[25] Roy U, Kornitzer D. Heme-iron acquisition in fungi. Curr Opin Microbiol. 2019;52:77–83. 31265986 10.1016/j.mib.2019.05.006

[26] Pinsky M, Roy U, Moshe S, et al. Human serum albumin facilitates heme-iron utilization by fungi. mBio. 2020;11:e00607–20. 32317324 10.1128/mBio.00607-20 PMC7175094

[27] Pihet M, Le Govic Y. Reappraisal of conventional diagnosis for dermatophytes. Mycopathologia. 2017;182:169–180. 27718160 10.1007/s11046-016-0071-y

[28] Clinical and Laboratory Standards Institute. Reference method for broth dilution antifungal susceptibility testing of filamentous fungi. CLSI Standard M38, 3rd ed. 2017; Wayne, PA.

[29] Song Y, da Silva NM, Weiss VA, et al. Comparative genomic analysis of capsule-producing black yeasts Exophiala dermatitidis and Exophiala spinifera, potential agents of disseminated mycoses. Front Microbiol. 2020;11:586. 32373085 10.3389/fmicb.2020.00586 PMC7179667

[30] Xiao C, Chen Y, Xie S, et al. MECAT: fast mapping, error correction, and de novo assembly for single-molecule sequencing reads. Nat Methods. 2017;14:1072–1074. 28945707 10.1038/nmeth.4432

[31] Lypaczewski P, Hoshizaki J, Zhang W, et al. A complete Leishmania donovani reference genome identifies novel genetic variations associated with virulence. Sci Rep. 2018;8:16549. 30409989 10.1038/s41598-018-34812-x PMC6224596

